# Hyperspectral retinal imaging in Alzheimer’s disease and age-related macular degeneration: a review

**DOI:** 10.1186/s40478-024-01868-y

**Published:** 2024-10-03

**Authors:** Xiaoxi Du, Jongchan Park, Ruixuan Zhao, R. Theodore Smith, Yosef Koronyo, Maya Koronyo-Hamaoui, Liang Gao

**Affiliations:** 1 Department of Bioengineering, University of California Los Angeles, Los Angeles, CA USA; 2 Ophthalmology, New York Eye and Ear Infirmary of Mount Sinai, New York, NY USA; 3 Department of Neurosurgery, Maxine Dunitz Neurosurgical Research Institute, Cedars-Sinai Medical Center, Los Angeles, CA USA; 4 Department of Neurology, Cedars-Sinai Medical Center, Los Angeles, CA USA; 5 Division of Applied Cell Biology and Physiology, Department of Biomedical Sciences, Cedars-Sinai Medical Center, Los Angeles, CA USA

**Keywords:** Hyperspectral imaging, Retinal imaging, Alzheimer’s disease, Neurodegenerative disease, Age-related macular degeneration

## Abstract

While Alzheimer’s disease and other neurodegenerative diseases have traditionally been viewed as brain disorders, there is growing evidence indicating their manifestation in the eyes as well. The retina, being a developmental extension of the brain, represents the only part of the central nervous system that can be noninvasively imaged at a high spatial resolution. The discovery of the specific pathological hallmarks of Alzheimer’s disease in the retina of patients holds great promise for disease diagnosis and monitoring, particularly in the early stages where disease progression can potentially be slowed. Among various retinal imaging methods, hyperspectral imaging has garnered significant attention in this field. It offers a label-free approach to detect disease biomarkers, making it especially valuable for large-scale population screening efforts. In this review, we discuss recent advances in the field and outline the current bottlenecks and enabling technologies that could propel this field toward clinical translation.

## Introduction

Neurodegenerative diseases such as Alzheimer’s disease (AD) exert substantial social and economic burdens on our aging population. With an estimated 6.9 million people living with AD in the United States and about 50 million people worldwide affected by AD and associated dementia, this number is projected to triple by 2050 [1, 2]. This age-related epidemic is particularly concerning for the elderly, as the incidence of Alzheimer’s dementia sharply increases after 65 years of age, impacting over 33% of individuals aged 85 and older.

The diagnosis of AD often occurs during the late stage when clinical symptoms of dementia become apparent. However, clinical studies demonstrate that well before these symptoms manifest, neuropathological indicators of the disease can be identified through techniques such as amyloid positron emission tomography (PET) imaging [3] or cerebrospinal fluid (CSF) assays [4, 5]. This preclinical stage of AD, occurring before substantial and irreversible brain damage, presents an opportunity for intervention with potential therapies aimed at halting or slowing disease progression.

Despite the increasing focus of clinical research on developing treatments and strategies to mitigate risk or slow disease progression in AD, there’s still a critical need for reliable and cost-effective clinical assessment and diagnostic tools. This need is especially pronounced in the early stages of the disease, where interventions are actively being developed. While amyloid PET neuroimaging and CSF testing present high utility as diagnostic biomarkers in clinical trials, their limited availability in the clinical setting, invasiveness, time-consuming procedures, and high costs restrict their widespread use across populations. Therefore, there’s an urgent demand for an early, noninvasive, and cost-effective tool that can identify AD biomarkers. Such a tool would greatly aid in detecting Alzheimer’s pathology during the mild cognitive impairment (MCI) stage and even in asymptomatic preclinical AD cases, significantly enhancing our ability to predict individuals at high risk of developing dementia on a larger scale.

**Fig. 1:**
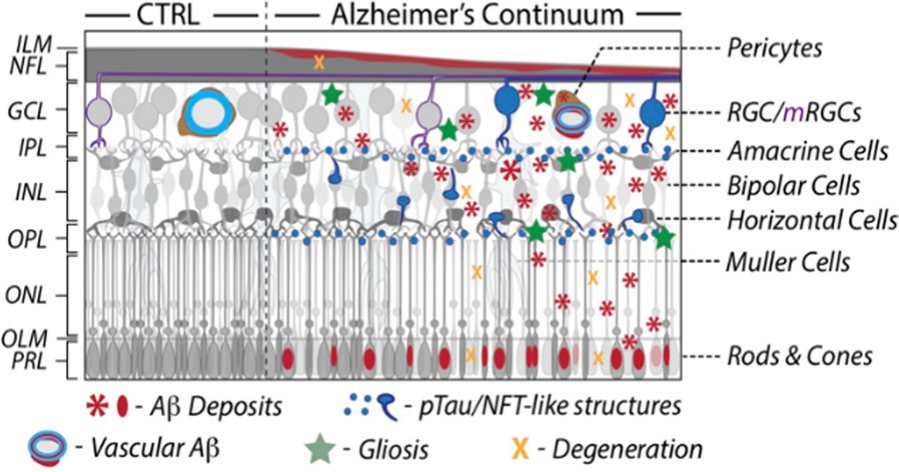
Schematic illustration of Alzheimer’s pathology across retinal cell layers in AD patients. CTRL, control. Illustration was adapted from Mirzaei et al., Frontiers in Neuroscience 2020 with permission [55]

One emerging technology that could address this demand is retinal imaging. Despite AD historically being viewed as a brain disorder, recent studies indicate its manifestation in the eye, with growing evidence of abnormalities in the retina, considered a sensory extension of the brain [6–30] (as illustrated in Fig. 1). Notably, hallmark AD pathological signs, such as amyloid β-protein (Aβ) and hyperphosphorylated (p)Tau protein (pTau) that is found in neurofibrillary tangles (NFTs), observed in the brain, have also been identified in the retina[17, 22, 31]. Increasing reports reveal abnormal Aβ and pTau deposits in the retinas of AD patients at various stages, distinct from non-AD controls [10, 11, 15–19, 22, 23, 26, 27, 32–54]. As the only central nervous system (CNS) tissue not protected by bone, the retina provides a unique opportunity to study neuro-pathological changes at the site of injury.

The evidence of Aβ and pTau accumulation in the retina at early AD stages supports the notion of using the eye for presymptomatic imaging. Notably, research by the Koronyo-Hamaoui group and others revealed retinal Aβ plaques, oligomers, and vascular Aβ deposits in preclinical, MCI, and early AD patients [10, 11, 16, 17, 26, 35, 46, 49, 56, 57] and in transgenic AD-model mice at the presymptomatic stage, preceding detection in the brain[17, 32]. Additional reports have shown pTau, tau oligomers, and other pathological tau isoforms in the retina of MCI and AD patients [15, 22, 27, 34, 37, 39, 42, 47, 51, 58, 59]. Given the direct and noninvasive nature of retinal imaging, detecting retinal AD pathology, particularly early Aβ and pTau biomarkers, may enable large-scale screening and monitoring of at-risk populations.

While imaging retinal Aβ and pTau deposits holds promise for early AD diagnosis, it presents challenges due to their similar visual appearance to normal tissue, resulting in low contrast with conventional fundus color photography. Recently, there has been a surge in interest in using hyperspectral imaging (HSI), which acquires both spatial and spectral information of the sample, to identify AD and neurodegenerative retinal biomarkers.

Initially designed for remote sensing applications, HSI has gained traction in various medical fields. The rationale behind employing HSI in medical imaging lies in the fact that the tissue’s intrinsic optical properties, like absorption and scattering, undergo changes during disease progression [60]. The light spectrum emitted from tissue carries quantitative diagnostic information about tissue pathology. Unlike conventional intensity-based cameras that capture only the two-dimensional (2D) spatial distribution of light, HSI records light in three dimensions (3D), simultaneously capturing spatial coordinates (x, y) and wavelengths (λ) of incident photons. This comprehensive dataset provides valuable insights for various medical diagnostics and interventions.

HSI is not only valuable for AD but has also proven effective in identifying spectral biomarkers associated with other neurodegenerative conditions like age-related macular degeneration (AMD). AMD is the leading cause of blindness in the world. The social and medical costs in the United States alone total billions of dollars annually [61]. AMD, a late-onset neurodegenerative retinal disease, shares key clinical and pathological features with AD, such as oxidative stress and inflammation responses to stress stimuli. Research indicates notable similarities in the intra- and extracellular deposits implicated in both diseases.

In this review, we discuss the recent advancements in hyperspectral retinal imaging and its relevance to neurodegenerative diseases, particularly AD and AMD. We believe these strides epitomize cutting-edge research in the field, showcasing significant progress that is reshaping early AD diagnosis approaches.

## Hyperspectral imaging data acquisition strategies

**Fig. 2:**
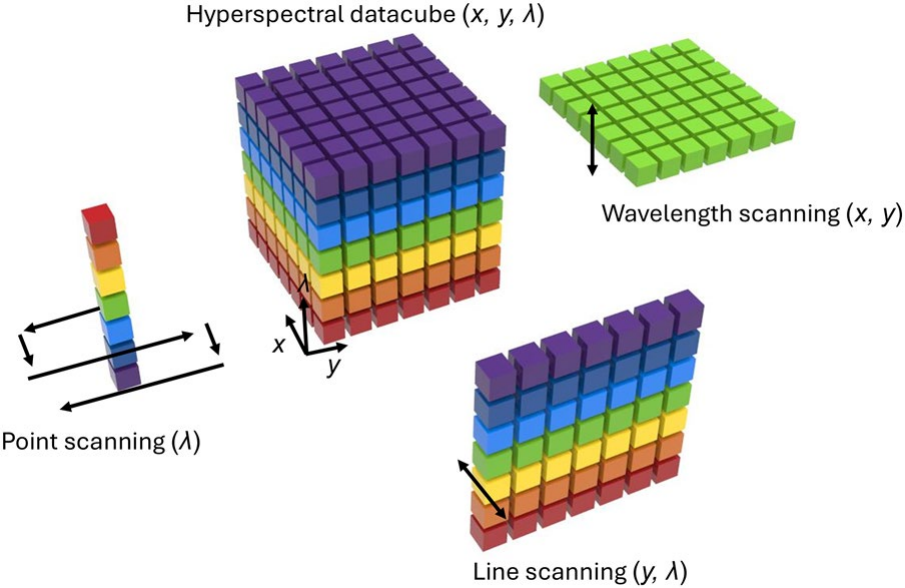
Hyperspectral imaging data acquisition strategies. Hyperspectral imaging systems generate a 3D hyperspectral datacube (*x, y, λ*). The point-scanning method uses a linear detector to collect spectral information (*λ*) from a single point in space. The line-scanning method captures a spatial-spectral slice (*y, λ*) of the datacube using a 2D detector. In the wavelength-scanning method, a 2D image (*x, y*) is captured at a specific wavelength. The snapshot method captures the entire 3D datacube (*x, y, λ*) in a single camera exposure

HSI acquires both spatial and spectral information of light, resulting in a 3D datacube (*x*, *y*, λ) [62, 63]. There are four distinct strategies for measuring this datacube, illustrated in Fig. 2. The first strategy, point-scanning HSI, utilizes a linear array of detectors to capture spectral information (λ) simultaneously, followed by scanning across all spatial locations (*x*, *y*) to construct the complete datacube. The second method, line-scanning HSI, uses a 2D detector array to capture one slice of the datacube (*y*, λ) at a time, requiring scanning in only one spatial dimension (*x*) afterward. Both point- and line-scanning HSI imagers necessitate extensive scanning during the acquisition of a large datacube. This results in prolonged acquisition times and makes them susceptible to motion artifacts when imaging dynamic scenes.

In contrast, wavelength-scanning HSI, the third approach, captures one slice of the datacube (*x*, *y*) and then scans across all wavelengths (λ). This simultaneous acquisition of spatial information results in significantly faster imaging speeds compared to point- or line-scanning methods. Examples of systems using this strategy include acousto-optic or liquid crystal tunable filter-based HSI systems [64]. The fourth strategy, snapshot HSI, captures the entire datacube in a single exposure, offering a means to completely eliminate motion artifacts, especially when combined with flash illumination. Technologies falling under this category include spectrally-resolved detector array (SRDC) [65], image mapping spectrometry (IMS) [66, 67], computed tomography imaging spectrometry (CTIS) [68], and coded aperture snapshot spectral imaging (CASSI) [69]. In the next section, we will delve into the detailed implementation of these strategies in retinal imaging.

## Hyperspectral retinal imaging: implementations

**Fig. 3:**
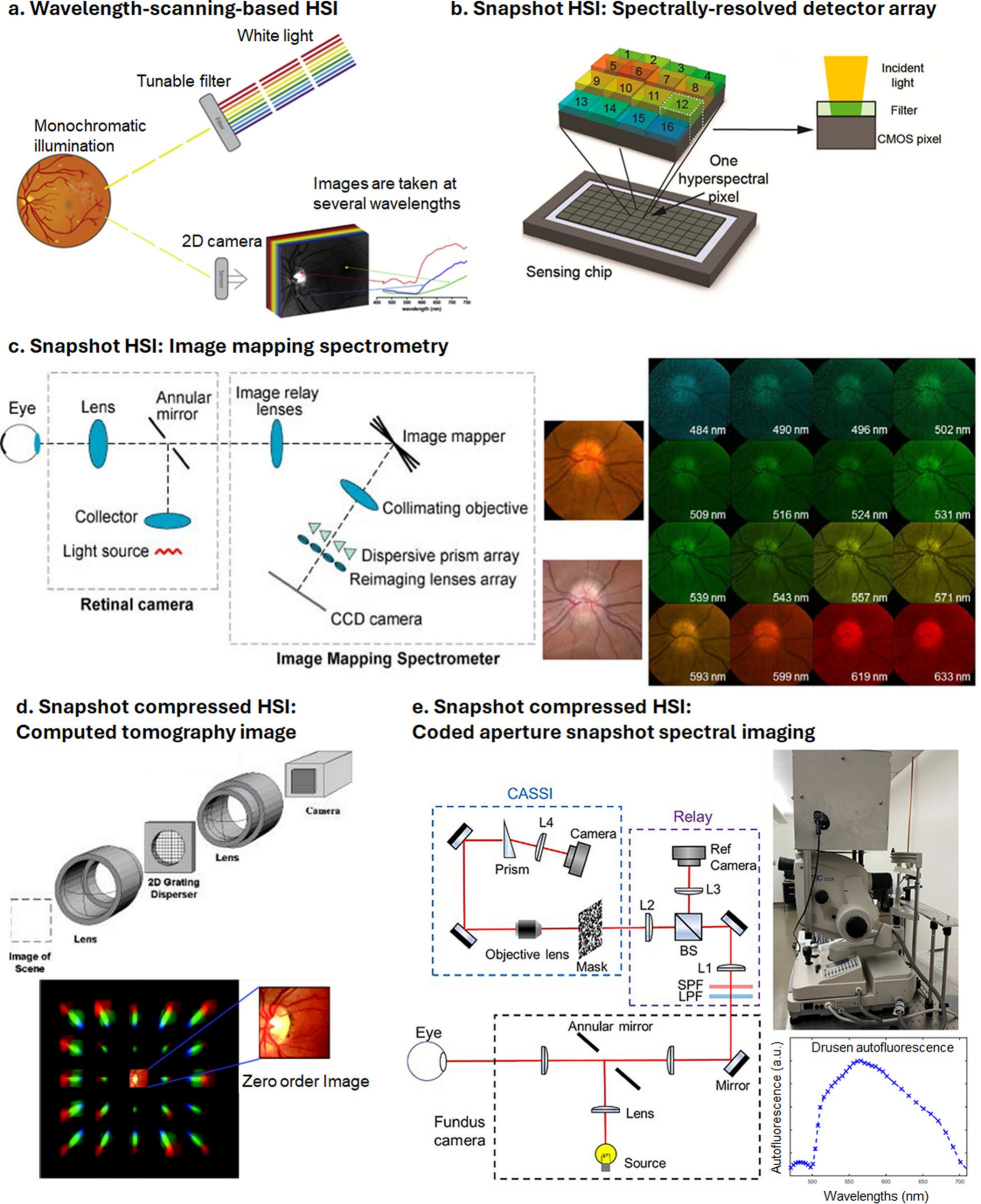
in vivo hyperspectral retinal imaging implementations for **a** Wavelength-scanning-based HSI. Retina is sequentially illuminated by monochromatic light with different wavelengths Desjardins et al. [70]. **b** Spectrally-resolved detector array. A thin-film Fabry–Perot cavity spectral filter is fabricated directly on each camera pixel. A total of 16 wavelength bands are achieved Li et al. [71]. **c** Image mapping spectrometry (IMS). (left panel) An image mapper consisting of multiple angled mirror facets slices and redirects the image to various regions of a detector. A prism array spectrally disperses the sliced images. (middle panel) IMS is combined with a fundus camera. (right panel) Snapshot hyperspectral imaging of human retina in vivo Gao et al. [72]. **d** Computed tomography imaging spectrometry (CTIS). A holographic grating diffracts the image in various directions Johnson et al. [73]. The hyperspectral datacube is reconstructed by using a tomographic image reconstruction algorithm. (right panel) Reconstructed hyperspectral images of macula with 75 spectral bands Fawzi et al. [74]. **e** Coded aperture snapshot spectral imaging (CASSI). (left panel) Optical system. The image is encoded with a random binary mask and dispersed by a prism Zhao et al. [75]. The image is reconstructed by solving the linear inverse problem associated with the image formation model. (right panel) Hyperspectral autofluorescence imaging of drusen in vivo. The spectrum is obtained by averaging the spectra over a drusen area (unpublished data). All panels are used with permission

HSI of the retina faces challenges due to the constant motion of the in vivo eye, necessitating shorter acquisition times to prevent motion artifacts. Consequently, point scanning or line scanning HSI systems are typically employed for ex vivo studies. For in vivo applications, the wavelength-scanning approach is favored for its faster acquisition and straightforward implementation (Fig. 3a). In these systems, a wavelength-scanning unit, such as a liquid–crystal or acoustic-optics tunable filter, can be placed directly before either the light source or the detector, allowing for easy integration with a standard fundus camera without extensive hardware modifications. It is important to note that while the unit functions similarly regardless of placement, positioning it before the light source is preferred to reduce the illumination dose on the retina.

There are several wavelength-scanning-based HSI retinal cameras designed for clinical applications. One such example is the Metabolic Hyperspectral Retinal Camera (MHRC) by Optina Diagnostics, tailored specifically for diagnosing retina-related diseases. The MHRC provides a 30-degree field of view, a pixel resolution of approximately 8.3 µm, and a spectral resolution of around 5 nm within the 450–900 nm spectral range. With an exposure time of 0.01 s per wavelength, it can capture the entire spectral range in under one second. The MHRC has been used in various retinal studies, including retinal oximetry [70, 76, 77], Aβ detection [24, 56, 78], and the identification of retinal ischemia [79] and hydroxychloroquine retinopathy [80].

Eliminating eye motion artifacts entirely would ideally involve conducting retinal hyperspectral imaging in a snapshot manner. However, snapshot HSI retinal cameras are often complex and have not seen widespread use in patient studies. The challenge arises from the ‘dimensionality gap’[81]: while a HSI datacube (*x, y, λ*) is 3D, most current image sensors are in a 2D format. To capture the spatio-spectral information in a hyperspectral datacube, the datacube voxels must be mapped to pixels on the camera sensor. Unlike sequential measurements used in scanning-based hyperspectral imagers, snapshot HSI employs more intricate methods to simultaneously measure light datacube voxels on a 2D detector array.

Snapshot HSI techniques can be categorized into two main methods based on their underlying principles. The first method, known as direct measurement, establishes a one-to-one mapping between light datacube voxels and camera pixels. By remapping the measured data, the light datacube can be reconstructed. Representative modalities employing this method include spectrally-resolved detector array (SRDA) and IMS.

The SRDA is a complementary metal-oxide semiconductor (CMOS)-based camera chip that employs pixel-wise filtering (Fig. 3b). This chip is structured on a CMOS architecture, with its pixels grouped into super-pixels, also known as HSI pixels, each comprising *N* × *N* CMOS pixels, thereby representing *N*^*2*^ wavelength channels. Within each CMOS pixel in an HSI pixel, a dielectric-thin-film Fabry–Perot (FP) cavity filter is monolithically integrated onto the surface. In the FP cavity, incident light undergoes interference with the reflected light, allowing only a specific resonant wavelength of light to pass through. This resonant wavelength is controlled by the thickness of the dielectric film. As a result, the camera sensor equipped with this technology can capture multiple spectral channel images in a single snapshot. Using an SRDA camera sensor, Li et al. developed a snapshot HSI fundus camera and showcased its effectiveness in performing retinal oximetry in live rats [71]. Subsequently, the same research group extended this technique to human retinal imaging [82]. The SRDA image sensor is now commercially available through IMEC.

Despite their snapshot acquisition capability, SRDA image sensors exhibit low light throughput due to the use of narrow-band filters on top of each CMOS pixel, limiting the amount of light received by each pixel. Additionally, there exists a trade-off between the number of spectral bands and spatial resolution. Increasing the spectral bands necessitates grouping more pixels into an HSI pixel, enlarging the super-pixel size and consequently reducing spatial resolution.

In contrast, the IMS allows snapshot HSI with a full light throughput without using filters. IMS achieves this by employing a custom-designed mirror, known as the image mapper, which consists of multiple angled facets redirecting different parts of an image to various regions on a detector array (Fig. 3c). This redirection of image slices on the detector array creates spaces between slices, allowing a prism or diffraction grating to spectrally disperse light orthogonally to the length of each image slice. Consequently, IMS captures a spectrum from every spatial location in the image within a single camera frame acquisition. The original image can then be reconstructed through a straightforward remapping of pixel information.

The unique tilt angles of the facets in the image mapper establish a fixed one-to-one correspondence between each voxel in the datacube and each pixel on the camera. This position-encoded pattern on the camera provides simultaneous spatial and spectral information within the image. As IMS directly captures object irradiance, no reconstruction algorithm is necessary, simplifying the process to produce image and data displays. Gao et al. demonstrated the integration of IMS with a standard fundus camera for retinal oximetry [72]. Subsequently, they showcased its application in imaging eyes affected by AMD in a separate study [66].

Direct measurement methods like SRDA and IMS encounter a fundamental limitation with a given camera sensor: the total number of hyperspectral datacube voxels acquired cannot exceed the total number of camera pixels due to the one-to-one mapping relation. In contrast, the second HSI strategy, referred to as compressed measurement, multiplexes signals from multiple hyperspectral datacube voxels and maps them to the same camera pixel, resulting in a higher detector utilization ratio. Representative techniques within this category include CTIS and CASSI.

In CTIS, a computer-generated holograph (CGH) is positioned at the conjugate plane of the aperture stop within an imaging system. Unlike conventional diffractive gratings that disperse light along a single dimension, a CGH can disperse light in two dimensions, resulting in various combinations of diffraction-order images captured by the camera (Fig. 3d). Each diffraction-order image represents the outcome of two sequential operations applied to the hyperspectral datacube of the object: first, shearing the wavelength axis towards the direction associated with the image’s diffraction order, and second, summing the intensities along the wavelength axis. By employing a multiplicative algebraic reconstruction algorithm, the object’s datacube can be reasonably estimated from these captured images.

The compact design of a CTIS camera facilitates its integration into a standard fundus camera setup. Johnson et al. exemplified [73] this integration by employing such a system for conducting retinal oximetry in live human subjects. In a later study, Fawzi et al. expanded on its application by demonstrating its effectiveness in mapping macular pigment in vivo [74]. However, CTIS faces a challenge known as the “missing cone” problem in the spatial-spectral frequence space, arising from the limited spectral dispersion power of the CGH and the finite area of the camera sensor. This limitation results in reduced spatial–temporal resolution in CTIS imaging.

In contrast, CASSI utilizes a conventional prism or grating to disperse the image and records a single spatial-spectrally multiplexed image (Fig. 3e). To mitigate the ill-posed nature of the image formation model, CASSI employs an absorption mask to encode the input image with a random binary pattern before spectral dispersion. Image reconstruction in CASSI involves solving the inverse problem of this image formation process, which can be achieved using algorithms like gradient projection for sparse reconstruction or a two-step iterative shrinkage/thresholding algorithm. Zhao et al. built a snapshot HSI fundus camera using this technology and demonstrated its application in in vivo retinal autofluorescence imaging of AMD patients (Fig. 3e, middle and right panels) [75].

While simpler in hardware compared to direct measurement approaches mentioned earlier, both CTIS and CASSI are computationally intensive. Their image reconstructions often depend on iterative algorithms, which are typically slow and lack immediate feedback. Additionally, their reliance on compressed sensing necessitates sample sparsity in specific domains, limiting their ability to extract complex spectral signatures of underlying chromophores. However, recent advancements in deep learning show promise in accelerating image reconstruction and enhancing image quality, offering a potential solution to these challenges [83].

## Hyperspectral retinal imaging: applications in neurodegenerative diseases

The overall rationale of using HSI in disease diagnosis is that the tissue’s endogenous optical properties, such as absorption and scattering, change during the progression of the disease, and the spectrum of light emitted from tissue carries quantitative diagnostic information about tissue pathology.

Two primary retinal biomarkers for AD are amyloid beta-protein (Aβ) and hyperphosphorylated (p)Tau, both known for their significant neurotoxic effects. The scattering spectral signature of Aβ was first identified ex vivo by More and Vince in 2015 [44]. Their study on retinal specimens from APP1/PS1 transgenic mice, a standard AD animal model, revealed notable differences in the scattering spectrum between 480 and 560 nm compared to wild-type mice, occurring months before observable amyloid plaques in brain tissue. Moreover, they showed that treatment with the anti-AD drug ψ-GSH caused a shift in retinal spectra towards the saline-treated wild type rather than the saline-treated transgenic mice, indicating potential therapeutic effects.

The reduction in spectral intensities at shorter wavelengths was postulated to be attributed to Aβ aggregates’ scattering properties, with their geometric size being much smaller than the visual light wavelength. Consequently, Rayleigh scattering predominated at Aβ aggregated sites, where light intensity inversely correlated with the fourth power of the wavelength. This led to greater attenuation of short-wavelength light passing through these locations, resulting in diminished intensities within the corresponding spectrum range. The same researchers later demonstrated a similar short-wavelength optical density increase both in in vivo APP/PS1 transgenic AD mice [84] and AD patients [57]. Particularly in patient imaging, they noted an inverse relationship between this optical density rise and Mini-Mental State Exam scores, highlighting the potential of this optical marker in clinical assessments of neurodegenerative diseases.

The unique scattering spectral signature of Aβ aggregates has also been independently reported by several other groups. Lemmens et al. [1] employed a snapshot hyperspectral retina camera to record reflectance spectra from both AD patients and control subjects. Their findings revealed a heightened optical density specifically within the wavelength range of 460–600 nm. Furthermore, they demonstrated that integrating this spectral characteristic with data on retinal nerve fiber layer thickness obtained through optical coherence tomography (OCT) can enhance diagnostic accuracy.

In another study, Hadoux et al. [56] reported a significant difference in reflectance spectra between individuals with high Aβ burden observed on brain PET imaging with mild cognitive impairment and age-matched controls. Specifically, the average reflectance spectrum from AD patients showed a decrease in wavelengths below 490 nm compared to controls. This discovery was corroborated in another cohort using a separate hyperspectral camera, reinforcing the reliability of these spectral differences. Similar variations in spectra were also observed between control subjects and 5xFAD transgenic mice known for Aβ accumulation in both brain and retina. Noteworthily, in this study, researchers emphasized the need to correct measured spectra for sources of spectral variability such as lens effects, macular pigment, melanin, and hemoglobin. Failure to make such corrections led to non-statistically significant differences between AD cases and controls.

**Fig. 4:**
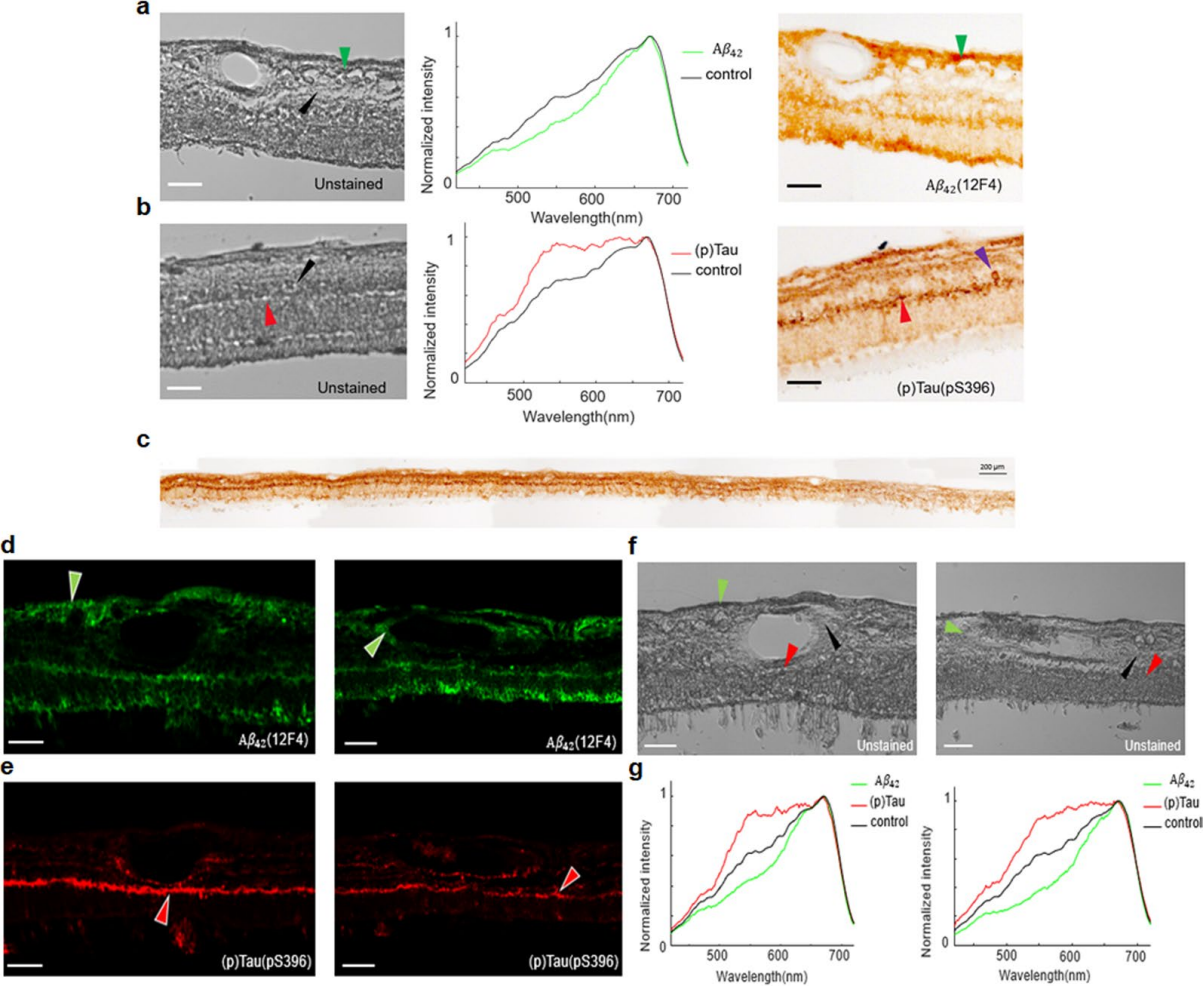
Hyperspectral imaging of Aβ_42_ and pS396-Tau deposits on postmortem retinal cross-sections of AD patients guided by a-c peroxidase-based immunostaining (DAB) and d-g immunofluorescence staining. **a**, **b** From left to right, unstained hyperspectral intensity images, spectra at arrow-pointed locations (green, red, and black arrows), and DAB-labeled images. The purple arrow (b, right) indicates a neurofibrillary tangle (NFT) structure in the OPL. Scale bar, 50 µm. **c** Tile image of a large portion of retinal cross-section strip from a confirmed AD patient immunolabeled for pS396-Tau DAB substrate. **d** Aβ_42_ immunofluorescence channel (green pseudocolored). **e** pS396-Tau immunofluorescence channel (red pseudocolored). **f** Unstained hyperspectral intensity images. **g** Spectra at arrow-pointed locations (green, red, and black arrows). Scale bar, 50 µm. Source: Du et al. with permission [34]

**Fig. 5:**
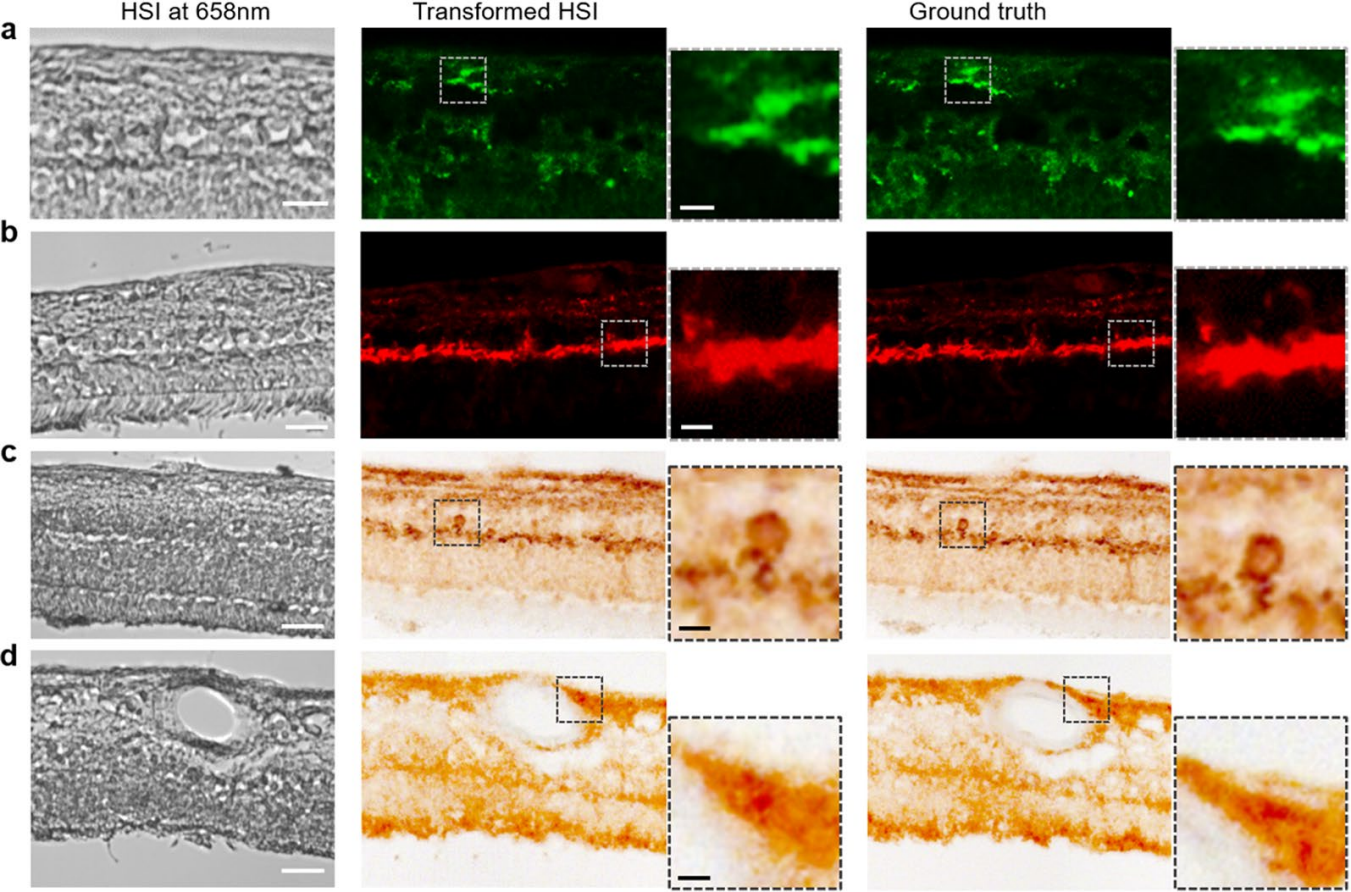
Machine learning prediction of Aβ_42_ and pS396-Tau based on HSI. **a** Aβ_42_ fluorescence model. **b** pS396-Tau fluorescence model. **c** pS396-Tau DAB model, with a focus on a retinal NFT structure. **d** Aβ_42_ DAB model. From left to right: HSI intensity image, transformed HSI images, zoomed prediction images of specific feature, ground truth images, and zoomed ground truth images of specific feature. Scale bar, 50 µm for large FOV images, 10 µm for bordered inserts. Microscope images adapted from Du et al. with permission [34]

Our research team has also detected the scattering spectral signature of Aβ_42_ aggregates, pathognomonic to AD, within retinal cross sections from postmortem AD patients by using a wavelength-scanning hyperspectral retinal camera [34]. What sets our study apart is we measured this spectrum within depth-resolved retinal layers, thereby minimizing the influence of non-AD ocular sources on spectral measurements. By directly comparing these findings with immunofluorescence ground truth, we observed a reduction in spectral intensities between 450 and 600 nm in retinal layers known to be rich in Aβ (nerve fiber layer, ganglion cell layer, outer plexiform layer, and outer nuclear layer) when compared to normal controls (Fig. 4). To translate this spectral discovery to chromophore mapping, we developed a deep-learning-based network capable of transforming label-free hyperspectral images into immunofluorescence and peroxidase-based immunostaining (also referred to as DAB) images. The resultant Aβ_42_ abundance map closely aligned with ground truths (Fig. 5).

Besides identifying the scattering spectral signature of Aβ_42_, we also observed the spectral signature of pTau. Employing a methodology akin to what we used for quantifying Aβ, we first immunolabeled pS396-Tau isoforms and identified their mostly aggregated locations in the retina, including outer plexiform layer, inner plexiform layer, and ganglion cell layer, and structures that resemble NFTs. We then examined these locations in the unstained HSI images acquired from adjacent retinal cross sections. Figure 4c illustrates the distribution of pS396-Tau deposits spanning from the central to peripheral retina. The pS396-Tau clusters showcase a distinct spectral profile that significantly deviates from that of typical retinal tissues—exhibiting notably higher and more consistent light transmittance in the 550–﻿650 nm range, akin to a “flat hat.” (Fig. 4b and 4g) This prominent characteristic suggests that tissues enriched with pTau have a diminished optical density within this spectral range, likely due to a lower absorption coefficient of their constituent chromophores. This led us to further scrutinize the HSI images at these wavelengths. Our observations revealed that pTau aggregation in the outer plexiform layer—which appears dark brown with DAB substrate and red in immunofluorescence-stained images—correlates with elevated pixel intensities in the grey-level HSI images (Fig. 4f). A similar correlation was also evident in the pS396-Tau aggregation area in NFTs (Fig. 4b), supporting our findings regarding the spectral transmission properties of pTau. Importantly, this marks the inaugural identification of pS396-Tau in the human retina.

Rather than relying solely on spectral information, Thach et al. [85] introduced a feature-extraction approach that combines spatial and spectral data from a wavelength-scanning reflectance hyperspectral retinal camera. Their method identified 30 significant spatial-spectral features essential for classifying cerebral PET amyloid status, providing a promising means to differentiate between PET amyloid positive and negative individuals. In another study, Sharafi et al. [24] reported a similar approach, and they extracted eight spatial-spectral features from retinal arterioles and their adjacent regions that proved effective in discriminating PET amyloid positive and negative subjects.

HSI has also proven effective in identifying spectral biomarkers associated with AMD. In early AMD eyes, soft drusen and basal linear deposits constitute the lipid-rich components of the “Oil Spill on Bruch’s membrane” [86]. While drusen are clinically identifiable focal deposits, basal linear deposits are thin, diffuse, and often invisible even on high-resolution OCT images but detectable through hyperspectral autofluorescence imaging [87]. This opens the possibility of the earliest possible detection of AMD with a clinical HSI fundus camera and treatment with antioxidant regimens proven to slow its progression[88]. For AMD eyes, a distinct short-wavelength spectrum (SDr) was first identified for drusen and sub-RPE deposits, emitting near 510 nm [89]. A subsequent study demonstrated that SDr exhibited both high sensitivity and specificity for identifying these AMD lesions [87].

**Fig. 6:**
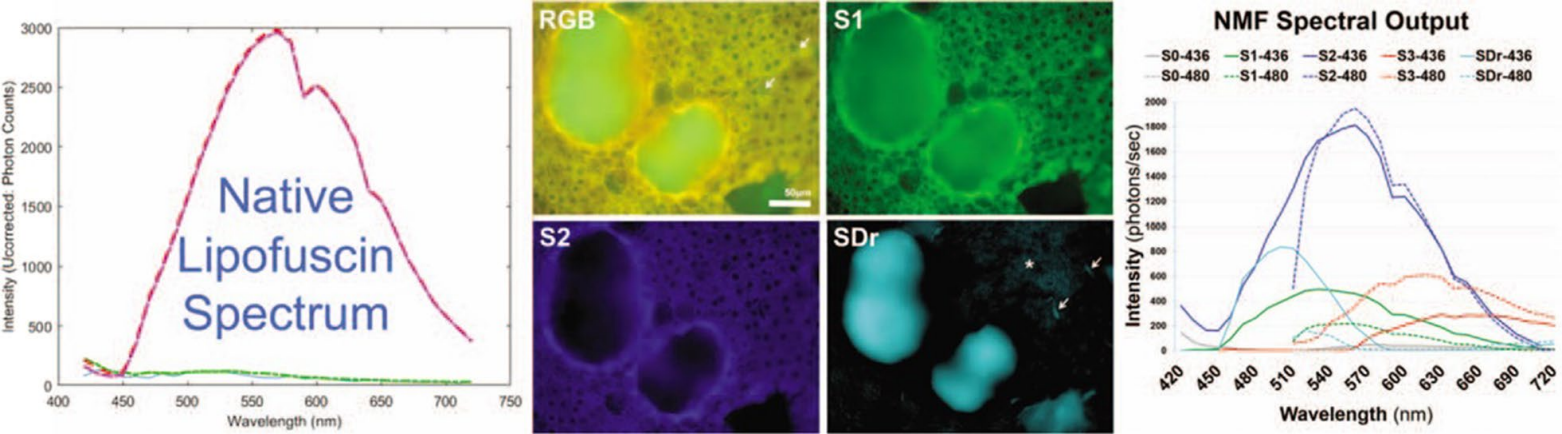
Hyperspectral Imaging of AMD Drusen The broad emission spectrum of RPE lipofuscin from a flatmount of RPE/BRM when excited by 436 nm light, captured by the Nuance camera, shows a peak around 570 nm, in the yellow range (left panel). The full-color autofluorescence (AF) of the sample with drusen, marked in RGB, highlights the predominantly yellow AF from the lipofuscin surrounding the nuclei in the RPE cells, while the AF from the soft drusen is greenish. After mathematical “unmixing” of the AF from the sample, three distinct spectra (S1, S2, S3) are found in the RPE, presented in green, blue, and red, with a new spectrum specific for drusen/drusen precursors (SDr) in azure, showing a short wavelength emission around 510 nm (right panel). The color-coded tissue localizations of the fluorophore sources of the spectra S1, S2, and SDr are shown (S3 not shown) (middle panel). Abbreviations: AF, autofluorescence; BRM, Bruch’s membrane; RGB, composite red–green–blue autofluorescence image; LF, lipofuscin; RPE, retinal pigment epithelium; SDr, spectrum for drusen. *Source* Orellana-Rios et al. with permission [92]

The possibility of early detection of AMD with HSI was explored more fully in a study by Mohammed et al. [90]. HSI techniques were employed to analyze the autofluorescence properties of lipofuscin fluorophores within the retinal pigment epithelium (RPE). Using a wavelength-scanning HSI camera, they captured spectral images ranging from 420 to 720 nm. Employing a tensor decomposition method [91] on the hyperspectral data, they extracted both the spectral signature and abundance of constituent fluorophores. Their findings revealed smooth, well-defined spectra and tissue abundances consistent with the known perinuclear localization of lipofuscin/melanolipofuscin in the RPE. They also confirmed the SDr for drusen and sub-RPE deposits, emitting near 510 nm (Fig. 6). A subsequent study demonstrated that SDr exhibited both high sensitivity and specificity for identifying these AMD lesions [87].

The spectral signature of drusen has been observed not only in the autofluorescence imaging but also in the reflectance imaging mode. In a study [93] conducted by Wang et al., they used a SRDC hyperspectral camera to image the drusen of the AMD patients. They then applied a machine learning classification approach to the HSI data acquired and demonstrated that HSI offers an improved accuracy and sensitivity in classifying drusen compared to standard fundus RGB images.

We have summarized the applications of HSI in neurodegenerative diseases discussed in this section in Table 1.

**Table 1:** Hyperspectral retinal imaging in Alzheimer’s and neurodegenerative diseases

Authors	Neurodegenerative disease type	HSI technology used	Sample type and size	Spectral range inspected	Major findings
More et al. [44]	AD	Line-scanning	Retinal flatmounts from APP/PS1 mice (n = 6) and AD patients (sample size unclarified)	400–1000 nm	Ex vivo spectral biomarker discovery of Aβ aggregates
More et al. [84]	AD	Wavelength-scanning	In vivo APP/PS1 mice (n = 8)	480–705 nm	In vivo spectral biomarker discovery of Aβ aggregates in AD mice
More et al. [57]	AD	Single-slit spectrograph	In vivo AD patients (n = 19)	400–1000 nm	In vivo spectral biomarker discovery of Aβ aggregates in AD patients
Sharafi et al. [24]	AD	Wavelength-scanning	In vivo AD patients (n = 20)	450–900 nm	Inclusion of metrics related to the retinal vasculature and tissue-related textures extracted from vessels and surrounding regions can improve AD diagnosis
Hadoux et al. [56]	AD	Wavelength-scanning	In vivo AD patients (n = 15) and in vivo 5xFAD mice (n = 12)	450–900 nm	Significant differences in the retinal reflectance spectra were found between individuals with high Aβ burden on brain PET imaging and mild cognitive impairment and age-matched PET-negative controls
Lemmens et al. [78]	AD	Spectrally-resolved detector array (SRDA)	In vivo AD patients (n = 17)	460–600 nm	Combined use of HSI and OCT provides enhanced accuracy for AD diagnosis
Du et al. [34]	AD	Wavelength-scanning	Ex vivo retinal cross sections from AD patients (n = 3)	420–720 nm	Ex vivo spectral biomarker discovery and validation of Aβ_42_ and pS396-Tau aggregates in AD patients
Thach et al. [85]	AD	Wavelength-scanning	In vivo AD patients (n = 25)	450–905 nm	Use of spatial-spectral features can improve the AD diagnostic accuracy
Mohammed et al. [90]	AMD	Wavelength-scanning	Ex vivo retinal flatmounts from AMD patients (n = 17)	420–720 nm	Ex vivo spectral biomarker discovery of drusen
Wang et al. [93]	AMD	Spectrally-resolved detector array (SRDA)	In vivo AD patients (sample size unclarified)	460–630 nm	In vivo classification of drusen
Tong et al. [87]	AMD	Wavelength-scanning	Ex vivo retinal flatmounts from AMD patients (n = 4)	420–720 nm	Ex vivo spectral biomarker discovery of drusen

## Discussion and perspective

A clinical retinal imaging system capable of detecting the earliest and most specific molecular markers of AD and other neurodegenerative conditions in vivo could serve as an invaluable early warning tool for at-risk individuals. It could also aid in the development and evaluation of new therapies based on a targeted molecular profile. Insights gained from studying presymptomatic individuals with AD pathology could significantly enhance our understanding of the disease’s onset in the retina, offering crucial guidance for early detection and potential cures. Additionally, the noninvasive, label-free imaging technique of HSI makes it particularly well-suited for large-scale population screening in routine office settings. This approach could extend significant health and social benefits to the aging population by enabling proactive intervention and management strategies.

Although current hyperspectral retinal imaging techniques for AD and other neurodegenerative conditions are advancing, they are still in their nascent stages, primarily focusing on spectral discovery and validation. In this section, we discuss existing bottlenecks and explore new avenues that could potentially expedite their clinical translation.

### Depth-resolved HSI

Although multiple studies have observed statistically significant spectral differences between patients affected by the neurodegenerative disease and age-matched controls, there remains considerable variability in the reported spectral data. For instance, while most studies indicate increased optical density in Aβ-deposit locations, a recent report shows the opposite—a reduced optical density [94].

One primary reason for these discrepancies is that much of the current HSI of the retina is performed through reflectance imaging, where the signals measured at the HSI image sensor reflect the integrated light attenuations along the light path. This approach is susceptible to variations caused by different ocular components contributing to these attenuations, along with their spectral characteristics varying from person to person and variability in Aβ-deposit in different anatomical regions of the retina. These factors collectively render the measured spectra sensitive to individual-specific variables.

To mitigate this problem and standardize retinal spectral measurement, we advocate for a transformative shift in methodology from conventional reflectance imaging to depth-resolved imaging. As indicated by our prior research, AD biomarkers such as Aβ and pTau aggregates within distinct retinal layers [34]. Additionally, factors like melanin within the RPE layer and the choroidal vascular bed can notably influence the spectral characteristics of reflected light. Thus, to accurately isolate the spectral signature of disease biomarkers, it is essential to conduct HSI in a depth-resolved manner.

Among existing depth-resolved retinal imaging techniques, spectroscopic optical coherence tomography (S-OCT [95–97]) stands out as particularly promising for this objective. Unlike conventional OCT, which primarily offers morphological and layer information, S-OCT enhances OCT’s capabilities to encompass both structural and molecular imaging with a straightforward post-processing approach. S-OCT leverages OCT’s interferograms to extract depth-resolved spectroscopic profiles of samples. This spectroscopic data allows for the identification of endogenous chromophores, imbuing OCT images with valuable molecular contrast.

### Multimodal imaging

Previous research has demonstrated that the progression of neurodegenerative diseases often coincides with structural and microvascular alterations in the retina. These changes include thinning of the inner retinal layer [98], choroidal layer [99], and peripapillary retinal nerve fiber layer [100], as well as reduced capillary density [101]. Such structural and microvascular changes have primarily been identified using high-resolution OCT and OCT angiography. Moreover, the accumulation of disease-specific molecules like Aβ on the retina has been visualized through noninvasive fluorescence imaging techniques employing the food additive curcumin [15]. A comprehensive review of these retinal imaging methods for AD and neurodegenerative diseases is available elsewhere [30].

However, a common limitation across current retinal imaging methods for diagnosing neurodegenerative diseases is their lack of specificity. For instance, structural changes like retinal thinning may indicate various conditions like glaucoma, diabetes, or inflammatory retinopathies, leading to diagnostic ambiguity. As different retinal imaging modalities provide unique structural and/or functional insights, integrating these modalities with HSI shows potential for improving diagnostic precision. This prospect is substantiated by insights gleaned from multiple pilot studies outlined in section “Hyperspectral retinal imaging: implementations”.

Given that the spectral signatures of neurodegenerative diseases discovered so far fall within the visible light range, the integration of visible OCT with HSI presents a compelling approach. Despite visual discomfort from visible light, visible OCT [102] has gained growing popularity in clinical settings due to its superior resolution compared to conventional near-infrared OCT systems. Importantly, visible OCT naturally aligns with HSI of retinal Aβ and other proteinopathies as its interferograms can directly provide spectroscopic information without requiring hardware modifications or additions. We anticipate that this synergy will enhance visible OCT’s capacity to detect biomarkers of neurodegenerative diseases, thus broadening its utility in this domain.

### Artificial intelligence

HSI often produces vast datasets, presenting a computational challenge for data processing. However, recent strides in artificial intelligence, especially deep learning, offer promising avenues to expedite HSI data processing and exploration. Within medical imaging, deep learning techniques have become increasingly favored for handling HSI images [103]. This preference stems from their exceptional ability to extract intricate spatial-spectral features from tissue, enhancing disease diagnosis and classification capabilities.

Deep learning models excel at discerning features from unstructured data, using these insights to make predictions based solely on data examples. One of the most prevalent deep learning architectures for image analysis is the convolutional neural network (CNN). When applied to retinal images of neurodegenerative disease patients, CNN models can identify known features described in scientific literature, uncover new observable features, and even detect subtle features beyond human perception [104]. This capability positions CNNs as highly promising tools for automating decision-making processes in neurodegenerative disease diagnosis using retinal HSI images, as evidenced in several pilot studies discussed in section “Hyperspectral retinal imaging: implementations”. Recently, commercialization efforts by RetiSpec [105] have been initiated, though their AI solution is currently available for research use only.

Another highly effective deep learning approach in processing hyperspectral retinal images is the generative adversarial network (GAN). A GAN comprises a generator and a discriminator, engaged in a competitive learning process. The discriminator is trained to differentiate between real inputs and those generated by the generator, enhancing its generalization capability, especially beneficial with limited training data. GANs excel in image transformation tasks. For instance, our prior research showcased its ability to convert label-free HSI retinal images into immunolabeled counterparts with remarkable fidelity, leveraging the spatial-spectral features within the HSI dataset [34]. This capability holds significant value as it enables the transformation of HSI images into clinically interpretable formats familiar to physicians, thereby expediting clinical translation.

A significant challenge in employing deep neural networks for hyperspectral retinal image analysis has been their black-box nature, lacking transparency in decision-making processes. Class activation mapping (CAM) offers a solution by pinpointing key features in either the original image or within convolutional layers that heavily influence a model’s decisions, particularly aiding in classification tasks [106]. The advantage of CAM lies in its potential to reveal meaningful clinical correlations of model behavior [107]. Despite these advancements, there is currently no established classification system for neurodegenerative diseases based on retinal hyperspectral image findings. Therefore, understanding the spatial-spectral features utilized by deep neural networks in decision-making processes will be crucial for connecting model predictions with physiological features, facilitating more insightful medical interpretations.

A notable recent approach in detecting and predicting diseases from retinal images is to use an ophthalmic foundation model [108, 109], a generalizable model trained using 1.6 million unlabeled retinal images. After fine-tuning, the foundation model can perform various downstream tasks, ranging from diabetic retinopathy screening to predicting progression of neurodegenerative disease. Incorporating HSI into such generalizable models will further improve the classification and prediction accuracy of various diseases, although a large number of hyperspectral retinal image sets in clinical settings is required. Furthermore, the inclusion of the additional wavelength dimension will lengthen the network development time and consumes significant computational resources.

## Data Availability

No datasets were generated or analysed during the current study.

## Abbreviations

AD: Alzheimer’s disease
PET: Positron emission tomography
CSF: Cerebrospinal fluid
MCI: Mild cognitive impairment
Aβ: Amyloid β-protein
pTau: Hyperphosphorylated (p)tau protein
NFTs: Neurofibrillary tangles
CNS: Central nervous system
HIS: Hyperspectral imaging
SRDC: Spectrally-resolved detector array
IMS: Image mapping spectrometry
CTIS: Computed tomography imaging spectrometry
CASSI: Coded aperture snapshot spectral imaging
MHRC: Metabolic hyperspectral retinal camera
SRDA: Spectrally-resolved detector array
CMOS: Complementary metal-oxide semiconductor
FP: Fabry–Perot
AMD: Age-related macular degeneration
CGH: Computer-generated holograph
OCT: Optical coherence tomography
RPE: Retinal pigment epithelium
SDr: Distinct short-wavelength spectrum
S-OCT: Spectroscopic optical coherence tomography
